# Increases in social support co-occur with decreases in depressive symptoms and substance use problems among adults in permanent supportive housing: an 18-month longitudinal study

**DOI:** 10.1186/s40359-020-00507-0

**Published:** 2021-01-06

**Authors:** Zhengqi Tan, Eun-Young Mun, Uyen-Sa D. T. Nguyen, Scott T. Walters

**Affiliations:** 1 Department of Biostatistics and Epidemiology, School of Public Health, University of North Texas Health Science Center, 3500 Camp Bowie Blvd., Fort Worth, TX 76107 USA; 2 Department of Health Behavior and Health Systems, School of Public Health, University of North Texas Health Science Center, 3500 Camp Bowie Blvd., Fort Worth, TX 76107 USA

**Keywords:** Permanent supportive housing, Social support, Depressive symptoms, Substance use problems, Latent growth modeling

## Abstract

**Background:**

Social support is a well-known protective factor against depressive symptoms and substance use problems, but very few studies have examined its protective effects among residents of permanent supportive housing (PSH), a housing program for people with a history of chronic homelessness. We utilized unconditional latent growth curve models (LGCMs) and parallel process growth models to describe univariate trajectories of social support, depressive symptoms, and substance use problems and to examine their longitudinal associations in a large sample of adults residing in PSH.

**Methods:**

Participants were 653 adult PSH residents in North Texas (56% female; 57% Black; mean age: 51 years) who participated in a monthly health coaching program from 2014 to 2017. Their health behaviors were assessed at baseline and tracked every six months at three follow-up visits.

**Results:**

Unconditional LGCMs indicated that over time, social support increased, whereas depressive symptoms and substance use problems decreased. However, their rates of change slowed over time. Further, in parallel process growth models, we found that at baseline, individuals with greater social support tended to have less severe depressive symptoms and substance use problems (coefficients: − 0.67, p < 0.01; − 0.52, p < 0.01, respectively). Individuals with a faster increase in social support tended to have steeper rates of reduction in both depressive symptoms (coefficient: − 0.99, p < 0.01) and substance use problems (coefficient: − 0.98, p < 0.01), respectively.

**Conclusions:**

This study suggests that plausibly, increases in social support, though slowing over time, still positively impact depressive symptoms and substance use problems among PSH residents. Future PSH programs could emphasize social support as an early component as it may contribute to clients’ overall health.

## Introduction

People who are homeless are at higher risk for health problems, such as malnutrition, stress, communicable diseases, and violence [1]. The elevated health risks among people who are homeless contribute to significantly higher mortality rates, shorter life expectancy, and more frequent hospitalizations and acute care services utilization, compared with people in the general population [2–4]. In particular, depression is approximately three times more prevalent among adults who are homeless (20–25%), compared with 8.1% among the general adult population of the United States (US) [2, 5]. Moreover, over a third of homeless individuals experience alcohol and drug problems, rates that are far greater than those seen among individuals with supportive housing [6]. Some evidence suggests that social support is negatively associated with both depressive symptoms and substance use problems among people who are homeless [7–12]. For example, in a sample of homeless women residing in a temporary shelter, marijuana use and drinking to intoxication were both predicted by lower social support [8]. Likewise, social support appears to be an important mediator of the effects of stress on depressive symptoms among people with limited resources [12]. Permanent supportive housing (PSH), which combines a housing voucher with supportive services, has been recognized as an effective model for stabilizing the mental and physical needs of homeless adults [1, 13]. Hence, the number of PSH beds in the US has increased by 380% or 144,000 more from 2007 to 2019 [14]. Despite this progress, a crucial knowledge gap remains: little empirical evidence exists to guide program decisions after homeless adults enter PSH. In particular, few studies have examined the longitudinal associations of social support with either depressive symptoms or substance use problems among PSH residents with a history of homelessness.

### Permanent supportive housing and social support

Though PSH residents are affected by many of the same issues that homeless individuals experience, PSH may provide the opportunity to begin new and steady social relationships and to gain support through newly formed connections [15]. For many people, it is a life-changing event. By providing stable housing and supportive case management services, PSH can be instrumental in breaking the negative reciprocal cycle that often exists between unstable housing, mental health, and substance use problems [16–19]. However, at the same time, PSH may also introduce new challenges to people who were once homeless [20, 21]. For example, some PSH residents experience social isolation after being housed in unfamiliar locations, while others feel stigmatized in environments without access to former peers [21]. However, very little longitudinal information on social support exists among the PSH residents. It remains an empirical question whether perceived social support increases in this population over time and whether changes in social support are related to health outcomes.

### Social support and depressive symptoms

In general, research suggests that depressive symptoms are negatively related to social support among people with current or past homelessness [22–24]. For instance, in two samples of homeless adults in a large metropolitan area, perceived social support buffered the effects of psychological stress [23]. In another sample of people residing in PSH, increases in social support over six months were associated with decreased depressive symptoms. However, most of the available evidence is either cross-sectional [23, 24] or focuses on temporally concurrent associations without examining the individual variability in underlying growth curves [22]. A growth perspective over time, as well as its limits or individual differences in the growth, may provide more helpful information for the purpose of improving care services. Given the heterogeneity in the course of adaptation and outlook for PSH residents, it may be important to study how changes in social support over time are associated with trajectories of depressive symptoms, while simultaneously estimating individual trajectories to assess their boundaries of promotive effects of one behavior over the other.

### Social support and substance use problems

Similarly, there is a dearth of information on the longitudinal relationship between social support and substance use problems among PSH population. A randomized trial of a “Housing First” PSH program on substance use problems among homeless individuals in Canada found an inconsistent effect of the housing intervention (vs. the treatment as usual group) on substance use problems [25]. Compared to the usual-care group, the treatment group experienced fewer substance-related problems after 12 months, but not after 24 months. Moreover, some substance use problems (e.g., relationship problems) decreased, while other problems (e.g., physiological tolerance) did not decrease over the 2 years following their housing placement [25]. In fact, findings from a few available studies suggest that substance use problems may continue even after housing has been provided [26, 27], and that more specific efforts to address stress and social support may be needed to help reduce substance use problems, especially if residents use substances to cope with stress [26]. The scant research on this population suggests that there are important knowledge gaps that need to be addressed—that is, whether substance use problems tend to decrease over time and whether a concurrent increase in social support plays a protective role.

### Measurement limitations of prior research

The existing research on PSH residents has often encountered measurement limitations. Many of the measures used in previous studies were adapted from scales developed for other purposes without empirical validation for applications to this specific population. For example, the measure of social support used by Durbin et al. [26] consisted of subscales from three different instruments designed for community residents with chronic mental illness, hospitalized patients with chronic psychiatric disorders, and the general US population [28–30]. A similar approach was employed in Kirst et al. [25], where they measured the substance use problems with items from one subscale of the Global Appraisal of Individual Needs Short Screener (GAIN-SS) [31], a screening tool designed for general populations. A recent psychometric study showed that this subscale might not be suited for people at low and high levels of severity [32].

The current study addresses these measurement weaknesses and provides a more rigorous test of the longitudinal relationships of social support, depressive symptoms, and substance use problems. We adapted the existing measures of social support and substance use problems for the PSH population: the Interpersonal Support Evaluation List (ISEL) [33, 34] and the Inventory of Drug Use Consequences (InDUC) [35]. To our knowledge, ISEL and InDUC have never been used or tested specifically in this population. Therefore, we modified the scales for this population and tested their psychometric properties in a series of confirmatory factor analyses (see Methods and Table 1). Using a latent variable modeling approach, we simultaneously tested measurement models for these three constructs over time and examined their parallel trajectories. Additionally, by simultaneously tackling measurement models and growth models in one analysis, we more efficiently use all available data and gain precision in estimation, while accounting for measurement errors, testing measurement invariance over time, and addressing missing data. We further included age, sex, race, and length of stay in the current neighborhood, as covariates to examine whether the initial levels of social support, depressive symptoms and substance use problems are explained by these covariates.

**Table 1:** Items measuring social support and substance use problems

A modified version of the Interpersonal Support Evaluation List (mISEL)		
Question	Thinking about the past month, how would you rate each of these items?	
Item number	Item	Subscale
1	I can easily find someone to help me think through problems	A
2	I can easily find someone to help me sort through my finances	A
3	I can easily find someone to give me advice when I need it	A
4	If I were sick, I could easily find someone to help me with daily chores	T
5	It could easily find someone who could give me a ride if I needed it	T
6	I could easily find someone to loan me $10 if I needed it	T
7	When I want to socialize, I have a group of friends I can spend time with	B
8	When I feel lonely, I have people I can talk to	B
9	I have a group of friends who include me in their activities	B

A modified version of the Inventory of Drug Use Consequences (mINDUC)		
Question	During the past 3 months, how often has this happened to you?	
Item number	Item	Subscale
1	My physical health was harmed by my drinking or drug use	P
2	My physical appearance was harmed by my drinking or drug use	P
3	I lost weight or didn't eat properly because of drinking or drug use	P
4	My family was hurt by my drinking or drug use	E
5	A friendship or close relationship was damaged by my drinking or drug use	E
6	My drinking or drug use damaged my social life, popularity, or reputation	E
7	I felt guilty or ashamed because of my drinking or drug use	R
8	I was unhappy because of my drinking or drug use	R
9	Drinking or drug use got in the way of my growth as a person	R
10	I took foolish risks while drinking or using drugs	I
11	While under the influence, I did impulsive things that I regretted later	I
12	I had an accident while I was under the influence	I
13	I spent too much or lost a lot of money because of drinking or drug use	S
14	I failed to do what was expected of me because of drinking or drug use	S
15	I had money problems because of drinking or drug use	S

Response options for social support are: 1 = hardly ever, 2 = occasionally, 3 = sometimes, 4 = most of the time, and 5 = almost always. Response options for substance use problems are: 0 = never, 1 = once or a few times, 2 = once or twice a week, and 3 = daily or almost daily

*A* appraisal, *T* tangible, *B* belonging; *P* physical, *E* interpersonal, *R* intrapersonal, *I* impulse control, *S* social responsibilities

We hypothesized that in the context of monthly health coaching, perceived social support would increase over time, whereas both depressive symptoms and substance use problems would decrease over time. We further hypothesized that individuals with lower levels of perceived social support at baseline would have higher levels of depressive symptoms and substance use problems at baseline and show slower rates of growth in social support over time, which would, in turn, predict slower rates of decline in depressive symptoms and substance use problems.

## Methods

### Mobile community health assistance for tenants

The current study used data from a health coaching program called “Mobile Community Health Assistance for Tenants” (m.chat). This program provided in-person health coaching to PSH residents as part of the Regional Healthcare Partnership (RHP 10) Medicaid Waiver program in the state of Texas (see more details of the m.chat program in [36]). In addition to the usual housing and case management services offered by the PSH programs, m.chat provided additional in-person health coaching to encourage PSH residents to adopt healthy behaviors, such as improved diet, exercise, recreation activities, or reducing substance use. The program consisted of monthly visits with a health coach who was trained in motivational interviewing and brief solution-focused therapy, a coaching software that provided feedback on health status, and access to wellness incentives (up to $60 in USD per month) that could be used to purchase health-related supplies. Health coaches began visits by eliciting areas of health concern and motivation for making changes. Clients discussed long-term health goals and developed short-term action items (using “SMART” goal planning). Coaches used the computer interface to set text reminders for clients who wanted to receive them and provided an overall summary of the visit. Follow-up visits included a review of recent progress (at the beginning) and discussion of how to use wellness incentives to achieve health goals (near the end). Coaching visits mostly took place in public locations, such as recreation centers, fast food restaurants, or the project office. Typically, participants completed one coaching visit per month for up to 18 months, and each visit lasted 52 min on average.

### Participants

Participants were 653 PSH residents (56% female; 57% Black; mean age: 51 years with a range of 20–80) recruited from six local housing agencies in Fort Worth, TX, from 2014 to 2017. Table 2 describes the sample and measures. In this sample of 653 people, 70% (n = 455) participated in the first follow-up (6 months after baseline), 46% (n = 299) in the second follow-up (12 months after baseline), and 38% (n = 249) in the third follow-up (18 months after baseline). All m.chat participants were Medicaid enrolled or low income uninsured, and self-reported one of the following mental health conditions in the past year: prescribed medication for psychological or emotional problems, experienced hallucinations, received a pension for a psychiatric disability, or scored greater than nine on a depressive symptoms screener, the Patient Health Questionnaire-9 (PHQ-9) [37]. Exclusion criteria included: (1) residing in other types of housing not considered PSH (e.g., Transitional Housing or homeless shelter), (2) any physical or sensory impairment that would substantially limit program participation, (3) non-English-speaking, or (4) limited autonomy or decision-making capabilities (e.g., substantially neurologically or cognitively impaired). This project was approved by the North Texas Regional Institutional Review Board.

**Table 2:** Descriptive statistics (*N* = 653)

Variables				
Age, mean (*SD*)	51.3	(10.0)		
Median (*IQR*)	53.0	(12)		
Sex	*n*	(%)		
Female	366	(56.1)		
Male	287	(43.9)		
Race/ethnicity				
White	212	(32.5)		
Black	371	(56.8)		
Hispanic	39	(6.0)		
Other	31	(4.7)		
Length of living in the current neighborhood				
Less than 1 year	286	(43.9)		
1–3 years	222	(34.1)		
More than 3 years	144	(22.0)		
Social support	*M*	(*SD*)	*n*	Range
BL	24.15	(8.72)	627	9–45
FU1	26.45	(9.20)	453	9–45
FU2	27.09	(9.27)	299	9–45
FU3	26.26	(9.51)	245	9–45
Depressive Symptoms				
BL	12.64	(6.31)	644	0–27
FU1	8.43	(5.74)	514	0–24
FU2	8.37	(5.81)	336	0–27
FU3	8.61	(5.93)	248	0–25
Substance use problems				
BL	5.25	(9.61)	636	0–42
FU1	3.86	(7.62)	506	0–42
FU2	3.85	(7.89)	332	0–43
FU3	4.47	(8.45)	249	0–43

*M* mean, IQR interquartile range, *SD* standard deviation, *BL* Baseline, *FU1* 6-month follow-up, *FU2* 12-month follow-up, *FU3* 18-month follow-up

### Measures

#### Social support

Social support was assessed with a modified version of the Interpersonal Support Evaluation List (ISEL) [33, 34]. The original ISEL measures perceived social support across four domains, each consisting of 10 items: (1) appraisal support, which is the perceived availability of other people to offer advice, guidance, and information, (2) tangible support, which includes aid or instrumental support, (3) self-esteem maintenance, which is the perceived availability of positive comparison to others, and (4) belonging support, which is the perceived availability of others for companionship [33, 34]. To simplify the language and make the examples more relevant to PSH residents, the ISEL was modified by removing the self-esteem maintenance domain, reducing the number of items in each domain from ten to three (nine items in total), and changing the scoring rubric. A series of statements about different aspects of social support (e.g., “When I feel lonely, I have people I can talk to,” “I could easily find someone to loan me $10 if I needed it”) were self-rated on a 5-point scale from “Hardly ever” to “Almost always” (Table 1). Cronbach’s alpha for the current sample was 0.87.

#### Depressive symptoms

Depressive symptoms were assessed with the Patient Health Questionnaire-9 (PHQ-9) [37]. The nine items on the PHQ-9 correspond to the nine criteria for major depressive disorder based on the Diagnostic and Statistical Manual of Mental Disorders-IV (DSM-IV) [38]. The items ask how frequently the individual has experienced each symptom during the last two weeks and assigns a score of 0, 1, 2, and 3 for not at all, several days, more than half of the days, and nearly every day, respectively. Items are summed for a total PHQ-9 score, which can be categorized as follows: mild if the resulting sum score ranges from 5 to 9, moderate for 10 to 14, moderately severe for 15 to 19, and severe for 20 to 27 [37]. Cronbach’s alpha for the current sample was 0.81.

#### Substance use problems

Substance use problems were assessed with a modified version of the Inventory of Drug Use Consequences (InDUC) [35]. The original version of the InDUC includes 50 items that evaluate both lifetime and recent (past three months) substance use problems from five domains: Physical, Interpersonal, Intrapersonal, Impulse Control, and Social Responsibility. In this study, the InDUC was modified by reducing the number of questions in each dimension from five to three and by asking participants only about recent (past three months) problems. Response options were: 0 = Never, 1 = Once or few times, 2 = Once or twice a week, and 3 = Daily or almost daily (see also Table 1). Cronbach’s alpha for the current sample was 0.96.

### Data analyses

We first conducted a confirmatory factor analysis to examine the structure and performance of social support, substance use problems, and depressive symptoms at each time point. We then used latent growth curve models (LGCM) to estimate changes in social support, depressive symptoms, and substance use problems at baseline, 6-month, 12-month, and 18-month post-baseline [39]. We tested several longitudinal measurement invariance models following recommendations and steps from Muthén and Christoffersson [40], Meredith [41], and Widaman and Reise [42] to ensure that item measures and latent traits are comparable across time. We started with the least restrictive measurement model (configural invariance) and moved to test stricter invariance models [41] by increasingly constraining parameters (factor loadings, intercepts, and residual variances) across time. We used the chi-square test of model fit to determine whether adding constraints to the model resulted in a poor fit. We selected the strictest invariance model with an acceptable fit.

We used the maximum likelihood estimator in the LGCM for social support and depressive symptoms and the robust maximum likelihood (MLR) estimator for the LGCM of substance use problems. The scaled difference chi-square test statistic [43] was calculated accordingly for models using MLR. We subsequently modeled the parallel trajectories [44, 45] of social support and depressive symptoms, and the parallel trajectories of social support and substance use problems in two separate, bivariate longitudinal analyses [46] to examine the relationships of the parameters from one trajectory with those from the other trajectory [47].

We estimated the parameters using all available information under the full information maximum likelihood (FIML) method for missing data [48]. The adequacy of model fit was examined using several absolute and relative fit indices, including the chi-square degrees of freedom ratio (χ^2^/*df*), the root mean squared error approximation (RMSEA) [49, 50], and the comparative fit index (CFI) [51]. Chi-square to *df* ratios less than 3; RMSEA scores less than 0.08; and CFI scores greater than 0.90 are generally considered acceptable [52–56]. All data preparation was conducted using SAS 9.4 (SAS Institute Inc., Cary, NC), and all factor analyses and latent growth curve modeling were conducted using Mplus 8.4 [57]. Figures 2 and 3 and all additional file figures were drawn using Microsoft PowerPoint.

## Results

### Measurement models: confirmatory factor analysis

Before fitting univariate growth curve models, we separately fitted measurement models for social support, depressive symptoms, and substance use problems at baseline and follow-ups (6-month, 12-month, and 18-month post-baseline). Table 2 provides a descriptive summary of the three health behaviors at the four assessment time points. All measurement models were fitted following the structure of the measurement tool for that health behavior (a 2^nd^-order, three-factor model for social support, a single-factor model for depressive symptoms, and a 2^nd^ order, five-factor model for substance use problems; see Additional file 1: Figs. S1–S12). Note that we allowed residual item variances to covary (see detail in Additional file 1: Figs. S1–S12, and measurement model fit indices for the three health behaviors across four waves in Additional file 1: Table S1). We kept these residual covariances in the subsequent univariate growth curve models. Overall, the model fit was adequate for all three behaviors, and all factor loadings were statistically significant, supporting the validity of the measurement models (full results available upon request).

### Univariate unconditional latent growth curve analysis

We tested up to the 2nd polynomial term in all growth curve models. Based on our prior experience, we anticipated that participants would have non-zero values at baseline that significantly vary across individuals. We further expected that all three behaviors could be modeled by a linear growth term and a quadratic growth term to capture their change over time. Table 3 presents the model fit information and the growth parameters of the univariate unconditional latent growth curve models of all three health behaviors. All three models fit the data adequately.

**Table 3:** Model fit and estimated parameters from the univariate unconditional growth models

	Model fit			Parameters					
Variable	χ^2^/df	RMSEA	CFI	Intercept	*SE*	Linear slope	*SE*	Quadratic slope	*SE*
Social support	1119.9/606	0.04	0.94	2.84**	0.05	0.34**	0.05	− 0.09**	0.02
Depressive symptoms	1020.8/563	0.04	0.92	1.49**	0.04	− 0.51**	0.07	0.12**	0.02
Substance use problems	2858.1/1724	0.03	0.90	0.28**	0.02	− 0.08**	0.02	0.02**	0.01

*SE* standard error

*p < 0.05; **p < 0.01

**Fig. 1:**
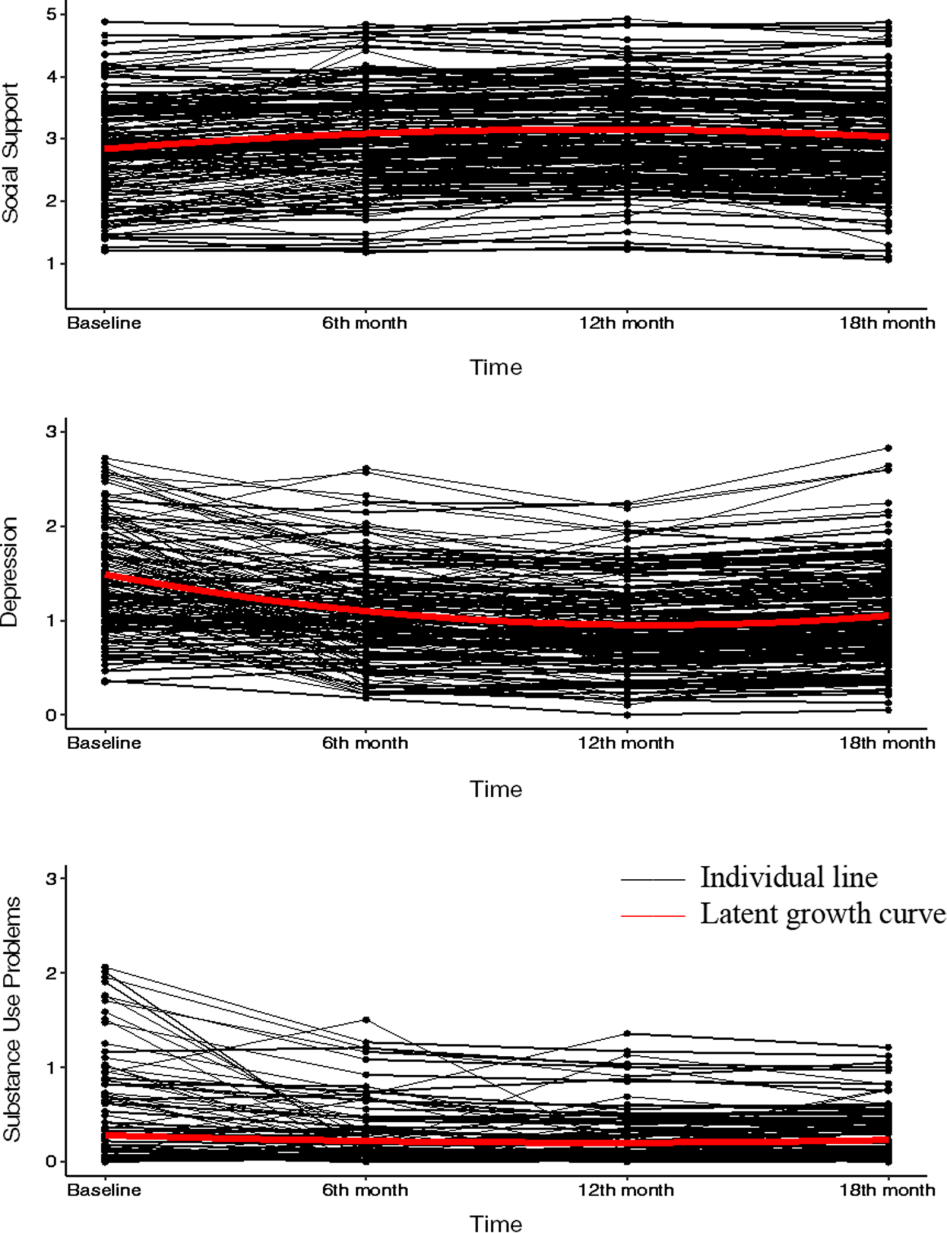
Estimated individual and average growth trajectories of social support (top), depressive symptoms (middle), and substance use problems (bottom) for a randomly selected 25% sample. Y-axis values indicate the average item score of each measure. Social Support: 1 = hardly ever to 5 = almost always. Depressive symptoms: 0 = not at all, 1 = several days, 2 = more than half of the days, and 3 = nearly every day. Substance Use Problems: 0 = never, 1 = once or few times, 2 = once or twice a week, and 3 = daily or almost daily

The intercept represents the average baseline level, the linear growth term quantifies the linear rate of change, and the quadratic term quantifies the rate of change in the linear growth rate over time. Social support significantly linearly increased (0.34) over time, with the *rate* of increase in social support slowing (− 0.09) over time (Fig. 1). For depressive symptoms, the negative linear growth term for depressive symptoms indicates that they significantly decreased (− 0.51) over time; however, the positive and statistically significant quadratic term indicates that reductions in depressive symptoms slowed (0.12) over time. For substance use problems, the negative linear growth term shows that they significantly decreased (− 0.08) over time, while the positive and statistically significant quadratic term indicates that the *rate* of reductions in substance use problems slowed (0.02) over time. We constrained the variances of quadratic growth terms to zero, specifying that any individual differences for the quadratic terms are trivial, which is a common observation in growth analysis [57]. Hence, there was no covariation between the quadratic terms and other growth parameters.

In testing measurement invariance across time points, the LGCMs of both social support and substance use problems held the strong factorial invariance in which invariance constraints were placed on the configural patterns, the factor loadings, and the intercepts of the measured item variables. The LGCM of depressive symptoms held the configural invariance, in which the pattern of fixed and free factor loadings of measured variables remained the same across time points.

### Parallel-process growth models

**Fig. 2:**
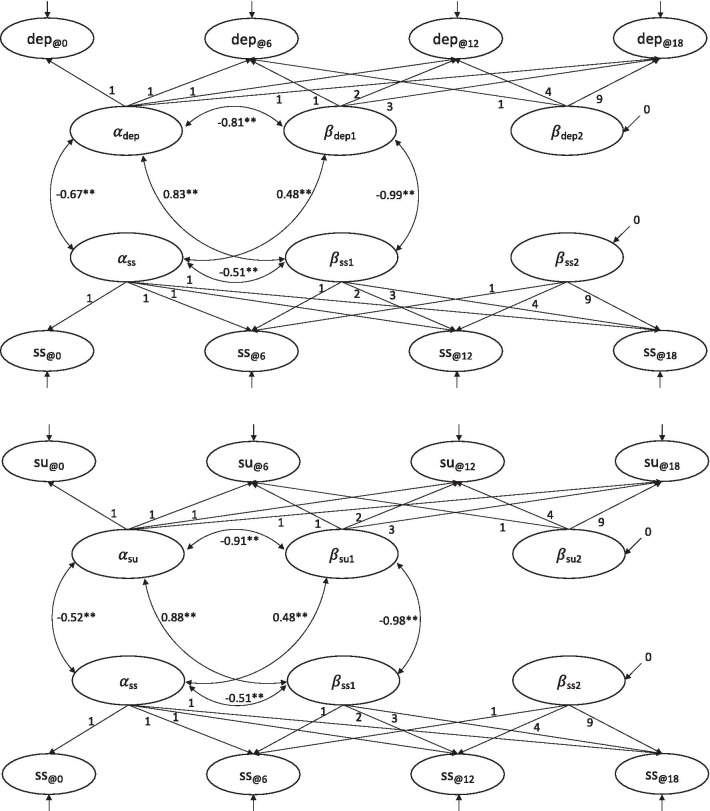
The unconditional parallel process growth curve models, examining the trajectories of social support and depressive symptoms (top), and social support and substance use problems (bottom). α_ss_: Intercept for social support; β_ss1_: Linear slope for social support; β_ss2_: Quadratic slope for social support; α_dep_: Intercept for depressive symptoms; β_dep1_: Linear slope for depressive symptoms; β_dep2_: Quadratic slope for depressive symptoms; α_su_: Intercept for substance use problems; β_su1_: Linear slope for substance use problems; β_su2_: Quadratic slope for substance use problems. ss@0-ss@18, dep@0-dep@18, and su@0-su@18 represent data over four observations for social support, depressive symptoms, and substance use problems. The residual variances (error variances) are not shown for better readability. *p < 0.05; **p < 0.01

The associations between the growth parameter terms for social support and depressive symptoms, and for social support and substance use problems were simultaneously assessed in two separate parallel-process growth models (Fig. 2). By setting the variance of the intercept and slope term to one, we directly estimated the correlation coefficients among them. The intercept-to-intercept, slope-to-slope, and intercept-to-slope correlation coefficients are presented in Table 4. The initial status of social support was negatively related to the initial status of both depressive symptoms and substance use problems. The intercept-to-slope associations within the same behavior domain showed the same patterns as those reported in the previous section on univariate unconditional latent growth curve analysis. Cross-domain correlations in the intercept and slope were statistically significant and large in magnitude. Higher levels of social support at baseline were related to lower levels of depressive symptoms (− 0.67) and substance use problems (− 0.52) at baseline. After controlling for the baseline differences and within-domain associations, the rates at which social support grew almost perfectly correlated with the rates at which depressive symptoms (− 0.99) and substance use problems (− 0.98) decreased.

**Table 4:** Estimated parameters and standard error (SE) of the unconditional parallel process latent growth models (also shown in Fig. 1)

	Estimate	*SE*
*Model: Social support and Depressive symptoms*		
Prospective association		
(Intercept-to-slope)		
Social support—social support	− 0.51**	0.05
Social support—depressive symptoms	0.48**	0.06
Depressive symptoms—social support	0.83**	0.03
Depressive symptoms—depressive symptoms	− 0.81**	0.02
(Intercept-to-intercept)		
Social support—depressive symptoms	− 0.67**	0.05
(Slope-to-slope)		
Social support—depressive symptoms	− 0.99**	0.01
*Model: Social support and Substance use problems*		
Prospective association		
(Intercept-to-slope)		
Social support—social support	− 0.51**	0.06
Social support—substance use problems	0.48**	0.08
Substance use problems—social support	0.88**	0.02
Substance use problems—substance use problems	− 0.91**	0.01
(Intercept-to-intercept)		
Social support—substance use problems	− 0.52**	0.07
(Slope-to-slope)		
Social support—substance use problems	− 0.98**	0.01

The estimates are standardized parameter estimates

*p < 0.05; **p < 0.01

**Fig. 3:**
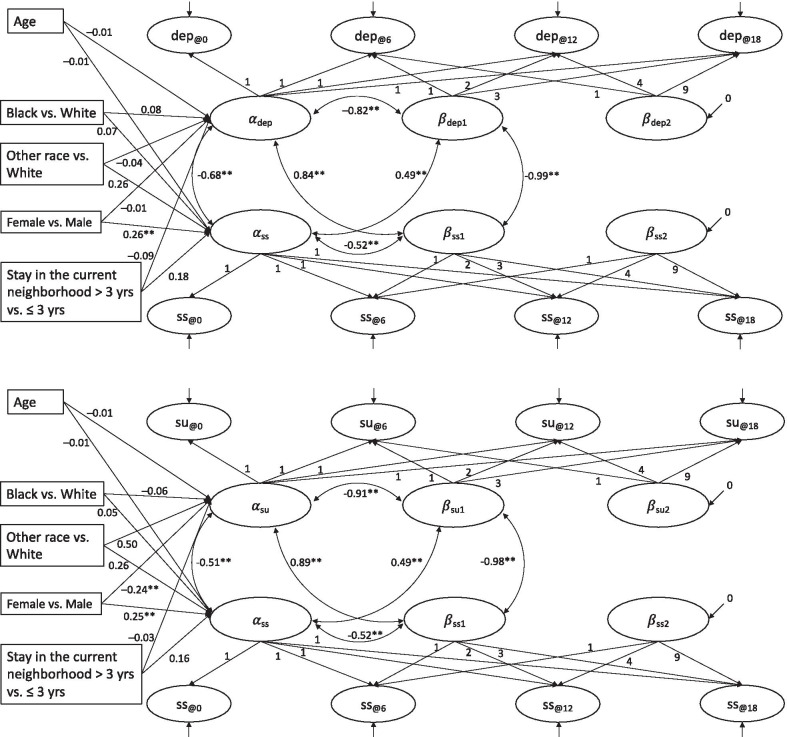
The conditional parallel process growth curve models, with the latent intercepts of social support and depressive symptoms (top), and social support and substance use problems (bottom) regressed on age, race, sex, and length of stay in the current neighborhood. α_ss_: Intercept for social support; β_ss1_: Linear slope for social support; β_ss2_: Quadratic slope for social support; α_dep_: Intercept for depressive symptoms; β_dep1_: Linear slope for depressive symptoms; β_dep2_: Quadratic slope for depressive symptoms; α_su_: Intercept for substance use problems; β_su1_: Linear slope for substance use problems; β_su2_: Quadratic slope for substance use problems. ss@0-ss@18, dep@0-dep@18, and su@0-su@18 represent data over four observations for social support, depressive symptoms, and substance use problems. The residual variances (error variances) are not shown for better readability. *p < 0.05; **p < 0.01

We added covariates, including age, sex, race, and length of stay in the current neighborhood, to examine whether the initial levels of depressive symptoms and substance use problems are explained by these covariates (see Fig. 3). Among all the covariates, women, compared to men, presented a significantly higher intercept level of social support in both models and a significantly lower intercept level of substance use problems. However, compared to the unconditional models, key growth parameters in the conditional models remained the same with a few negligible changes.

## Discussion

The current study examined the trajectories of social support, depressive symptoms, and substance use problems in a sample of adult PSH residents over 18 months after entering a health coaching program. Over 18 months, significant improvements were observed in all three health behaviors. Specifically, perceived social support increased, whereas depressive symptoms and substance use problems decreased over time, although the rate of positive changes slowed over time. This is an encouraging finding, given that PSH residents have a greater need for physical and mental health services when providing adequate professional care could be costly [4]. With monthly brief health coaching lasting for about an hour or less, residents’ social support increased along with concurrent reductions in depressive symptoms and substance problems. Health coaching may be a relatively inexpensive and feasible way to promote healthy behavior change among PSH residents who are motivated to change their health behaviors.

More specifically, the findings highlight a sizable variability in perceived social support at baseline, with higher perceived social support associated with less severe depressive symptoms and lower levels of substance use problems. The baseline associations suggest that it may be important for PSH case managers to consider the degree of social support received by their clients. As shown in this study, baseline social support was significantly correlated with the indicators of mental health and substance use problems. This may help case managers to develop need-based customized case management plans.

Furthermore, we found that the PSH residents with low social support at baseline showed increasing social support over time. The data show their rate of growth in social support was faster than those with higher levels of social support at baseline. In addition, PSH residents showed steeper rates of reduction in depressive symptoms and substance use problems when their social support increased at faster rates, as we observed a negative association between the slopes of the trajectory of social support and depressive symptoms and the trajectory of social support and substance use problems. The strong cross-domain correlations in the linear slopes suggests that the benefit of improving social support among PSH residents may provide non-disorder-specific overall support for behavior change and improved psychological well-being.

To the best of our knowledge, this is the first study to examine the association between rates of social support and depressive symptoms, as well as between social support and substance use problems in a PSH sample using parallel-process growth models. Evidence from this study provides a strong rationale for improving social support for PSH residents with mental health and substance use problems. Because m.chat was not an experimental study, it is difficult to determine if positive changes were due to health coaching, other PSH supportive services, or natural changes over time. Nevertheless, the current study suggests that coaching and increased social support can help initiate positive changes in other areas of health.

We also note that this is the most extensive longitudinal study of PSH residents participating in a health coaching program. The results suggest several important implications for the future design of supportive housing programs. First, programs should include regular follow-ups to assess the status of related health behaviors among residents. Since improvements in social support and depressive symptoms may not be consistent over time, frequent regular assessment follow-ups may help case managers identify any emerging challenges or difficulties and offer corresponding services. Follow-up intervals may need to be even shorter than every six months, which we had in this study, to more accurately pinpoint the timing of an inflection where growth slows and consequently plan for an additional coaching session or short booster sessions, delivered briefly over phone calls or text messages, to empower their agency in health-promotive behaviors and further reinforce newly adopted healthy behaviors. Second, future programs could emphasize social support as an early component as it may bring a favorable prognosis on mental health and substance use outcomes. The findings from the current study further emphasize the importance of understanding the status and needs of social support among PSH residents, as stated in the classic work of Cohen and Wills [58]. The need for social support may be different based on the specific difficulties or stressors that people try to cope with [58]. Thus, a baseline social support survey is crucial to understand the specific support that PSH residents will need the most.

Similar directions of correlation between the growth pattern of social support and substance use problems were observed in the other parallel process model. As expected, the statistically significant negative link between the baseline social support and the linear changing rate of substance use indicates that PSH residents who had higher perceived social support at baseline experienced faster improvements in substance use problems. Similarly, the negative link between the baseline substance use problems and the changing rate of social support indicates that PSH residents who had more severe substance use problems at baseline experienced a slower increase in social support. Some researchers have speculated that social support and substance use might be mutually exclusive strategies to cope with stress, such that greater social support might suppress substance use problems [8]. The findings from the current study may reiterate the protective association between social support and substance use problems and emphasize the necessity of understanding the social support among PSH residents from the perspective of improving substance use problems. In the future, program officers might consider ways to strengthen social connections for people newly placed in PSH, for example, by encouraging social support groups, matching residents with a peer mentor, or considering the proximity of a person’s natural support networks when determining the location of supportive housing placement.

Our findings should be interpreted with several cautions. First, there are significant variations among healthcare and case management services, demographic distributions, and their interest in participating in health coaching programs within the PSH population. Because all participants in this study received monthly health coaching, it is challenging to attribute their positive changes solely to health coaching. Second, it is also possible that PSH residents with improving health may report greater social support, although the reported interpretation of the association between social support and health behaviors is more consistent with the literature. Considering the findings from past studies indicating that PSH programs alone may not be sufficient to improve social support among PSH residents [20, 21], we believe that an experimental or quasi-experimental study is needed to confirm and verify the effects of social support on depressive symptoms and substance use problems in this population over time. Third, measures of social support and substance use problems in this study were modified from the original versions, though they were all formally tested in the current study. Fourth, this was a study conducted in one US metropolitan area with limited diversity/heterogeneity in samples, methods, and coaching programs. Therefore, how well the findings from this study can be generalized is an open question. Finally, we had infrequent health outcomes assessment by a team of assessors every six months at 6, 12, and 18-months following baseline. Future studies using shorter and more frequent intervals, especially early during coaching, would support more fine-grained analysis and evidence to help guide PSH programs.

## Conclusions

This study is one of the few longitudinal studies to examine social support, depressive symptoms, and substance use problems among PSH residents, an underserved population. With relatively frequent follow-ups over 18 months, this study reveals the pattern of change in two sets of health behaviors simultaneously using parallel process models. The use of latent variable models, including measurement models and latent growth curve models, allows us to formally test our measurement tools to assure good performance and stable properties across time as well as to maximize the available data to examine the research questions. The findings in this study suggest that the trajectory of social support is very likely to influence the trajectories of depressive symptoms and substance use problems. These findings suggest new ways to integrate these behavioral health targets in the development of intervention and prevention programs for PSH residents.

## Supplementary Information

**Additional file 1**. Supplemental table for measurement model fit indices and diagrams for measurement models and latent growth curve models.

## Data Availability

The datasets used and analyzed during the current study cannot be made publicly available due to IRB restrictions, but may be available from the corresponding author on reasonable request.

## Abbreviations

PSH: Permanent supportive housing
LGCM: Latent growth curve model
m.chat: Mobile community health assistance for tenants
MLR: Robust maximum likelihood
RMSEA: Root mean squared error approximation
CFI: Comparative fit index
PHQ-9: Patient Health Questionnaire-9
InDUC: Inventory of drug use consequences
ISEL: Interpersonal support evaluation list
