# Supplementary material for: Increases in social support co-occur with decreases in depressive symptoms and substance use problems among adults in permanent supportive housing: an 18-month longitudinal study
Source: BMC Psychol. 2021 Jan 6;9:6. doi: 10.1186/s40359-020-00507-0 (PMC7789599; doi:10.1186/s40359-020-00507-0)
Supplement: Supplementary file 1 — Additional file 1. Supplemental table for measurement model fit indices and diagrams for measurement models and latent growth curve models. [file 40359_2020_507_MOESM1_ESM.docx]

| Table S1. *Measurement Model Fit Indices across Time Points* | | |  |  |
| --- | --- | --- | --- | --- |
| Variables | χ^2^/df | RMSEA | | CFI |
| Social Support |  |  | |  |
| BL | 44.1/21 | 0.04 | | 0.99 |
| FU1 | 35.0/23 | 0.03 | | 0.99 |
| FU2 | 30.8/22 | 0.04 | | 0.99 |
| FU3 | 42.5/23 | 0.06 | | 0.99 |
| Depressive Symptoms |  |  | |  |
| BL | 50.6/23 | 0.04 | | 0.98 |
| FU1 | 56.1/25 | 0.05 | | 0.97 |
| FU2 | 54.1/24 | 0.06 | | 0.97 |
| FU3 | 48.7/23 | 0.07 | | 0.97 |
| Substance Use Problems |  |  | |  |
| BL | 225.5/77 | 0.05 | | 0.98 |
| FU1 | 325.5/79 | 0.08 | | 0.96 |
| FU2 | 228.6/73 | 0.08 | | 0.97 |
| FU3 | 170.0/70 | 0.08 | | 0.97 |

*Note*. BL = Baseline, FU1 = 6-month follow-up, FU2 = 12-month follow-up, and FU3 = 18-month follow-up.

*Figure S1.* Measurement model of social support at baseline. The oval indicates latent variables; the rectangle indicates observed variables (items). SS = social support, @0 = baseline.

*Figure S2.* Measurement model of social support at 6-month post-baseline. The oval indicates latent variables; the rectangle indicates observed variables (items). SS = social support, @6 = 6-month post-baseline.

*Figure S3.* Measurement model of social support at 12-month post-baseline. The oval indicates latent variables; the rectangle indicates observed variables (items). SS = social support, @12 = 12-month post-baseline.

*Figure S4.* Measurement model of social support at 18-month post-baseline. The oval indicates latent variables; the rectangle indicates observed variables (items). SS = social support, @18 = 18-month post-baseline.

*Figure S5.* Measurement model of depressive symptoms at baseline. The oval indicates latent variables; the rectangle indicates observed variables (items). PHQ = Patient Health Questionnaire, @0 = baseline.

*Figure S6.* Measurement model of depressive symptoms at 6-month post-baseline. The oval indicates latent variables; the rectangle indicates observed variables (items). PHQ = Patient Health Questionnaire, @6 = 6-month post-baseline.

*Figure S7.* Measurement model of depressive symptoms at 12-month post-baseline. The oval indicates latent variables; the rectangle indicates observed variables (items). PHQ = Patient Health Questionnaire, @12 = 12-month post-baseline.

*Figure S8.* Measurement model of depressive symptoms at 18-month post-baseline. The oval indicates latent variables; the rectangle indicates observed variables (items). PHQ = Patient Health Questionnaire, @18 = 18-month post-baseline.

*Figure S9.* Measurement model of substance use problems at baseline. The oval indicates latent variables; the rectangle indicates observed variables (items). SU = substance use problems, @0 = baseline.

*Figure S10.* Measurement model of substance use problems at 6-month post-baseline. The oval indicates latent variables; the rectangle indicates observed variables (items). SU = substance use problems, @6 = 6-month post-baseline.

*Figure S11.* Measurement model of substance use problems at 12-month post-baseline. The oval indicates latent variables; the rectangle indicates observed variables (items). SU = substance use problems, @12 = 12-month post-baseline.

*Figure S12.* Measurement model of substance use problems at 18-month post-baseline. The oval indicates latent variables; the rectangle indicates observed variables (items). SU = substance use problems, @18 = 18-month post-baseline.

*Figure S13.* Latent growth curve model of social support. The oval indicates latent variables. SS = social support, @0 = baseline, @6 - 18 = 6- to 18-month post-baseline. *p < .05

*Figure S14.* Latent growth curve model of depression. The oval indicates latent variables. DEP = depression, @0 = baseline, @6 - 18 = 6- to 18-month post-baseline. *p < .05

*Figure S15.* Latent growth curve model of substance use problems. The oval indicates latent variables. SU = substance use problems, @0 = baseline, @6 - 18 = 6- to 18-month post-baseline. *p < .05
